# The Assessment of Pentraxin 3: A Novel Biomarker in Early Detection of Infection in Newborns

**DOI:** 10.1155/2021/6638622

**Published:** 2021-06-30

**Authors:** Małgorzata Baumert, Piotr Surmiak, Martyna Szymkowiak, Agnieszka Janosz

**Affiliations:** Department of Neonatology, Faculty of Medical Sciences in Katowice, Medical University of Silesia in Katowice, Poland

## Abstract

**Introduction:**

As the clinical manifestation of neonatal infection is nonspecific and characterised by varied clinical features, it is highly problematic to establish an early diagnosis. Recently, hopes have been raised by the new acute-phase protein—pentraxin 3 (PTX3). PTX3 belongs to the family of long pentraxins, which is synthesized in numerous cells like endothelial cells, macrophages, and monocytes infiltrating sites of inflammation. *Material and Methods*. In our research, we have enrolled 29 newborns with infection as the study group and 47 healthy ones as the control group, as well as their mothers. The C-reactive protein (CRP), procalcitonin (PCT), and PTX3 levels were determined in venous blood samples from all investigated neonates and their mothers. Moreover, PTX3 concentrations were assessed in the umbilical cord.

**Results:**

There were statistically significant differences in PTX3 levels between healthy and sick newborns both in the umbilical cord (*p* = 0.02) and venous blood (*p* = 0.01). The highest PTX3 concentrations were observed in children with infection in the presence of premature rupture of membranes (PROM). PTX3 concentrations in this group were significantly higher compared to those in healthy children without PROM. We observed elevated PCT levels in newborns with infection. No differences in CRP levels in 12 hours of life were noticed between the investigated groups. A comparison of ROC curves for PTX3 and PCT concentrations revealed similar sensitivity and specificity in the prediction of infection in neonates.

**Conclusions:**

Serum PTX3 is an important and specific biomarker of early infection. It is already elevated in the umbilical cord, so measuring PTX3 concentration might be useful in the early prediction of infection in newborns.

## 1. Introduction

Neonatal infection, due to its varied clinical manifestation and highly problematic early diagnosis, is one of the leading causes of mortality and morbidity in an early adaptive period [1, 2]. This diagnosis in neonates is usually based on frequently repeated laboratory tests (white blood cell count with the immature-to-total ratio, C-reactive protein and procalcitonin levels) with accompanying clinical manifestation.

However, there is still a need for a novel biomarker that could be used as a specific predictor in early diagnosis of newborns' infection [3]. A new acute-phase protein called pentraxin 3 (PTX3) has a potential to fulfill those requirements. Pentraxin 3 belongs to the family of long pentraxins, which is synthesized in numerous cells of the body, under the influence of proinflammatory factors (interleukin 1*β* (IL-1*β*), tumor necrosis factor (TNF-*α*)) in the presence of lipopolysaccharide or fragments of pathogens (outer membrane protein in Gram-negative bacteria) as well as *toll-like* receptor agonists [4, 5].

An increase in serum PTX3 level was observed as early as in the 1^st^ hour after the occurrence of proinflammatory signals, reaching the maximum value between the 2^nd^ and 6^th^ hours [6].

Additionally, only few studies about the usefulness of pentraxin 3 in an early prediction of neonatal infection have been reported recently, and therefore, further investigations are warranted [7].

## 2. Aim of the Study

The study was aimed at determining whether pentraxin 3 levels, measured in the umbilical cord as well as neonatal venous blood samples taken within 12 hours after delivery, could be used as a valuable marker in the early detection of infection.

## 3. Material and Methods

This prospective, case-control study was conducted in the Department of Neonatology, Faculty of Medical Science in Katowice, Medical University of Silesia in Katowice, in the period between January and December 2018. The study was approved by the appropriate Bioethics Committee (no. KNW/0022/KB1/97/I/17). All the parents provided a signed informed consent before recruitment into the research.

### 3.1. Study Population

In this study, we have enrolled 76 newborns (29 of them diagnosed with infection and 47 healthy ones) delivered by a cesarean section and their mothers. All of the recruited patients were from the Caucasian population.

The data on the subject demographics, clinical history, and relevant comorbidities were collected upon enrollment, including in particular the incidences of premature rupture of membranes (PROM) and *Streptococcus agalactiae* (GBS) colonization in their mothers (Table 1).

Based on clinical manifestation (poor suckling, lethargy, altered muscle tone, respiratory distress, apnea, poor perfusion, cyanosis, bradycardia, thermoregulatory disorders including fever or hypothermia, food intolerance, and metabolic acidosis) and laboratory test results (blood culture for aerobic/anaerobic bacteria, CRP, PCT, platelet count, and white blood cell (WBC) count), the infection was determined in neonates. On diagnosis of this neonatal condition, a standard 7-day course of antibiotics compliant with ward protocol (ampicillin and gentamicin) was included in treatment. Patient care and monitoring were performed as a part of the hospital's standard protocol.

For this study, we gathered 2 mL samples of the maternal venous blood collected immediately before delivery and 1 mL samples of the umbilical artery blood during the delivery. Additionally, blood samples (0.5 mL) from newborns were collected from the peripheral vessels during a routine blood sampling procedure in the first 12 hours of life.

In all systemic blood samples, levels of PTX3, CRP, and PCT as well as platelet and white blood cell (WBC) count were determined. Moreover, PTX3 concentrations were assessed in the umbilical cord.

The obtained serum was separated and stored at −80°C until every sample was collected.

Serum pentraxin 3 levels were assessed by an enzyme-linked immunosorbent assay (ELISA) kit (BioVendor; Brno, Czech Republic). The detection limit of the assay was 22 pg/mL.

The C-reactive protein levels were measured using the immunoturbidimetry method on Beckman Coulter AU analyzer and the procalcitonin concentrations with electrochemiluminescence method (ECLIA) using the Cobas E 601 analyzer, similar to our previous study [8].

### 3.2. Statistical Analysis

The Shapiro-Wilk test was used to verify the normal distribution of the collected data. The categorical variables were compared using the chi-squared test. The quantitative variables were presented as medians with 95% confidence intervals (95% CI), and the qualitative variables were given as percentage values. Since PTX3 concentrations in blood samples were not normally distributed, nonparametric tests were used for analyses. The continuous variables were compared using the Kruskal-Wallis test with post hoc analysis as well as the Mann–Whitney *U* test.

Moreover, Spearman's rank correlation was calculated for the laboratory measurements and clinical variables.

Predictive performance was assessed employing the area under the receiver operating characteristic (ROC) curve. The ROC curves and area under the curves (AUC) with the Youden index were used to assess the diagnostic accuracy of serum PTX3, PCT, and CRP levels for predicting the infection in the study group.

Statistical analysis was based on standard procedures available in STATISTICA 13.3 (StatSoft Polska Inc.) and MedCalc Software 12.7.4. Statistical inferences were based on significance level *p* ≤ 0.05.

## 4. Results

### 4.1. Demographic and Clinical Characteristics of the Study Population

In this study, 76 newborns were enrolled, of whom 41 were late preterm (born between the 34^th^ and 36^th^ weeks of gestation) and 35 of them were full term. We have started our analysis by demonstrating a lack of differences in PTX3, PCT, and CRP levels between full-term and late preterm newborns, presented in Table 2.

Due to the clinical manifestation and results of additional laboratory testing, 29 newborns were included as the study group (diagnosed with infection) and 47 as the control group (healthy ones).

There were statistically significant differences in PTX3 values in umbilical cord blood between healthy newborns and those with the infection (*p* = 0.02). Elevated concentrations of PTX3 and PCT were observed in the venous blood of the study group as compared to controls (PTX3, *p* = 0.01; PCT, *p* = 0.02). There were no statistically significant differences in CRP levels between the analyzed populations (*p* = 0.20), as presented in Table 3. The other assessed parameters (WBC, platelets, and the number of positive blood cultures) did not reveal any significant differences between groups (Tables 1 and 3).

**Figure 1:**
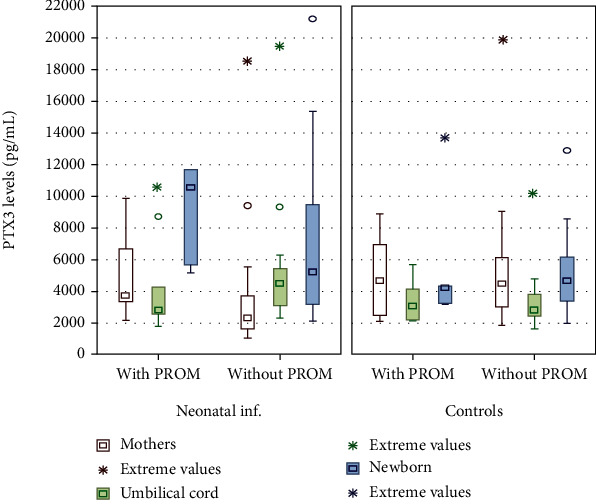
Serum pentraxin 3 levels in umbilical cord and venous blood from neonates with infection as well as healthy controls and their mothers with the prevalence of premature rupture of membranes (PROM). Results presented as a median as well as 95% confidence intervals for median and extreme values^∗^. ^∗^Newborns with both infection and PROM vs. neonates with infection and without PROM vs. controls; *p* = 0.02.

In venous blood samples, the highest PTX3 concentration was observed in sick neonates from pregnancies complicated by PROM. Regardless of PROM occurrence, the differences between investigated groups were statistically significant (*p* = 0.01), as seen in Figure 1.

### 4.2. Spearman's Rank Correlations

The entire research revealed a positive correlation between PTX3 levels and the incidence of PROM (rho = 0.24; *p* = 0.04) in the study group. In the first 12 hours of life, we observed a poor correlation between PTX3 and PCT concentrations (rho = 0.16; *p* = 0.05) in all newborns (Table 4).

### 4.3. The Receiver Operating Characteristic Curve Analysis

**Figure 2:**
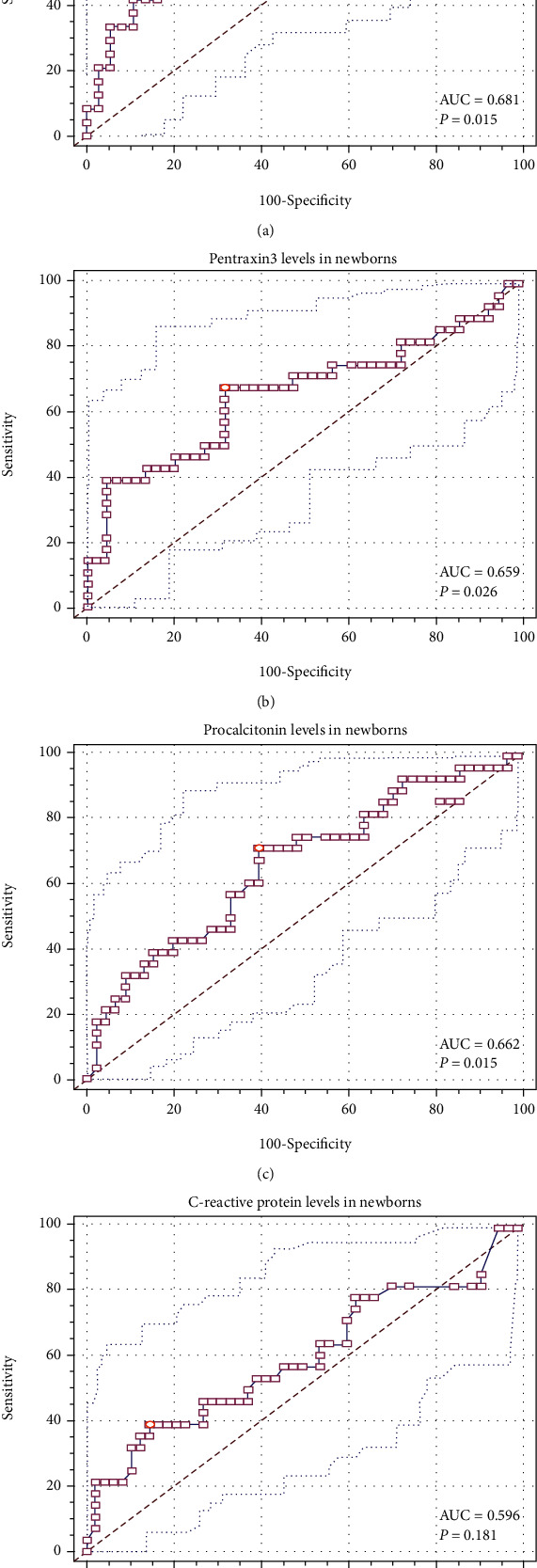
Receiver operating characteristic (ROC) curve with the Youden index for the (a) umbilical pentraxin 3 concentrations and (b) neonatal pentraxin 3 and (c) procalcitonin and (d) C-reactive protein levels in the early detection of infection in newborns.

The area under the receiver operating characteristic (ROC) curve for pentraxin 3 levels in umbilical cord blood was 0.681 (95% confidence interval (CI) 0.549-0.794), presented in Figure 2(a). Pentraxin 3 value in the umbilical cord of 3969.1 pg/mL was established as a cutoff value with 58.3% (95% CI 33.6-77.9) sensitivity and 78.4% (95% CI 61.8-90.2) specificity in prediction of infection in the first 12 hours of life.

The area under the ROC curve for pentraxin 3 levels in newborns' venous blood was 0.765 (95% confidence interval (CI) 0.65–0.86). The cutoff value for PTX3 was established at 8571 pg/mL with 61.1% (95% CI 78–99) sensitivity and 96.3% (95% CI 59–98) specificity in the early detection of this clinical condition (Figure 2(b)).

A similar result was obtained from the analysis of the receiver operating characteristic curve for PCT concentration at 12 hours of life in the prediction of infection in the study population (AUC 0.662) (95% CI 0.54–0.77; *p* = 0.02) (Figure 2(c)).

An analysis of the ROC curve for CRP levels in the first 12 hours of life revealed no statistical significance for the diagnosis of infection in the investigated group (*p* = 0.18) (Figure 2(d)).

A comparison of receiver operating characteristic curves for PTX3, PCT, and CRP concentrations did not demonstrate any statistically significant differences in the prediction of infection in newborns (*p* = 0.40) (Figure 2(e)).

## 5. Discussion

Researchers are still looking for markers that would allow an early diagnosis of the infection in newborns as it is crucial to avoiding health deterioration, reducing mortality, and preventing unnecessary use of antimicrobial agents.

So far, C-reactive protein (CRP) has been one of the most frequently used markers for diagnosing and monitoring inflammation in newborns. However, determining CRP levels has proven to have some limitations, especially when detecting an early infection in newborns. Berger et al. considered this protein to be a “specific,” albeit late marker of infection and inflammation [9].

C-reactive protein is the prototype of the short pentraxin subfamily. CRP can activate the classical complement pathway, stimulating phagocytosis and binding to immunoglobulin receptors (FcR). In humans, CRP levels may rise rapidly and markedly, after an acute inflammatory stimulus, largely reflecting increased synthesis by hepatocytes [10].

The pentraxin family, which includes CRP protein, is composed of short and long pentraxins. Pentraxin 3 has been classified as a member of the long pentraxin subfamily.

PTX3 is mainly produced at extrahepatic sites by several cell types and secreted at the site of inflammation, not only by endothelial cells but also by macrophages and monocytes infiltrating sites of inflammation [11, 12]. PTX3 is distinguished by being an early and local acute-phase biomarker, differing from CRP in gene organization, protein oligomerization, and expression pattern [13].

For this research, we assessed three acute-phase proteins (CRP, PCT, and PTX3) in both study groups. We concluded that there is a statistically significant difference in PTX3 levels in umbilical cord blood in newborns with infection as compared to healthy ones.

These findings may reflect an increased expression and production of PTX3 in the amniotic epithelium, chorionic mesoderm, trophoblast terminal villi, and perivascular stroma of human placentas [14].

During the first 12 hours of life, PTX3 concentration was significantly higher in newborns with infection as compared to healthy ones (*p* = 0.01). Similar findings were reported by other authors [15, 16]. However, in our study, there were no differences in PTX3 levels found, in regard to the occurrence of positive blood cultures in the studied group as compared to the controls. Perhaps this fact is related to the limited number of newborns with infection included into the studied group (*n* = 4; 13.8% of newborns with infection).

While studying the impact of PROM on PTX3 level, the authors found the highest marker levels in neonates with both infection and PROM. Additionally, a positive correlation between these factors was found. However, there were no significant differences in PTX3 concentrations between mothers, regardless of the occurrence of PROM. Akin et al. showed similar results in their study. On the other hand, Cruciani et al. presented different findings. The authors showed higher PTX3 maternal plasma concentration in preterm PROM, which constitutes part of the pathological activation of the inflammatory response in maternal circulation [17].

Procalcitonin is one of the analyzed markers that might rise rapidly in the course of infection in the first hours of life. Accordingly, we observed significantly higher PCT concentrations in the first 12 hours of life in newborns with infection in comparison to healthy ones, which was similar to the results reported by other authors [18]. We have identified the differences in PCT levels in neonates compared to their mothers in both study and control groups as presented in Table 3. In available manuscripts, the cutoff values of this biomarker were significantly higher in neonates than in the adult population, regardless of their clinical state [3, 19].

In our study, a cutoff value for PCT concentration above 8.01 ng/L was associated with sensitivity of 71.4% and specificity of 60.0% in prediction of the infection in newborns. The receiver operating characteristic curve for serum PTX3 level of 8571 pg/mL presents a moderate sensitivity (39.3%) and high specificity (95.5%) in the early prognosis of infection in the study group. However, there were no significant differences observed in the prediction of infection based on the comparison of ROC curves for PTX3 and PCT concentrations (*p* = 0.40). Thus, we have proposed that both factors could be used as independent and early infection markers in neonates.

In conclusion, serum PTX3 is an important and specific biomarker of early infection. It is already elevated in the umbilical cord, so measuring serum PTX3 might be useful in the prediction of infection in newborns.

## Figures and Tables

**Table 1:** Demographic data and perinatal characteristics of the study group (neonates with infections) and the control group (healthy newborns) and their mothers. Results are presented as medians and 95% confidence intervals or percentage values.

Investigated group	Study group (*n* = 29)	Control group (*n* = 47)	*p* value
Mothers			
(i) Age (years)	28 [24–31]	26 [23–30]	0.41
(ii) Primigravida (no.; %)	17; 58.6%	29; 60.4%	0.78
Perinatal history			
(i) Hypertension (no.; %)	3; 10.3%	9; 18.8%	0.31
(ii) Diabetes (no.; %)	4; 13.8%	8; 16.7%	0.38
(iii) Premature rupture of membrane (no.; %)	8; 27.6%	6; 12.5%	0.04
(iv) *Streptococcus agalactiae* colonization (+) (no.; %)	5; 17.2%	7; 14.5%	0.34

Neonates			
(i) Gestational age (weeks)	37 [35–38]	37 [36–39]	0.54
(ii) Gender: female (no.; %)	10; 34.5%	22; 45.8%	0.34
(iii) Birth weight (g)	2570 [2240–2970]	2605 [2350–3020]	0.45
(iv) Apgar 1^st^ min (no.; %)			
0–3 pts	2; 6.9%	4; 8.5%	
4–7 pts	4; 13.8%	3; 6.4%	
8–10 pts	23; 79.3%	40; 85.1%	0.54
(v) Apgar 5^th^ min (no.; %)			
0–3 pts	0	0	
4–7 pts	5; 17.2%	7; 14.9%	
8–10 pts	24; 82.8%	40; 85.1%	0.78
Postnatal complication			
(i) Fetal growth disturbances (no.; %)	5; 17.2%	9; 18.8%	0.86
(ii) Perinatal asphyxia (no.; %)	6; 20.7%	7; 14.5%	0.47
(iii) Thermoregulatory disturbance (no.; %)	12; 41.4%	8; 16.7%	0.03
(iv) Respiratory insuf. (no.; %)	15; 51.7%	9; 18.8%	0.02
(v) Circulatory insuf. (no.; %)	8; 27.6%	0	0.04
(vi) Positive blood cultures (no.; %)	4; 13.8%	0	0.53

**Table 2:** Serum pentraxin 3 (pg/mL), C-reactive protein (CRP) (mg/L), and procalcitonin (PCT) (ng/mL) levels in umbilical cord and venous blood from late preterm and full-term newborns as well as their mothers. Results are presented as medians and 95% confidence intervals.

Investigated group	Late preterm (*n* = 41)	Full term (*n* = 35)	*p* value
Mothers			
(i) PTX3 levels (pg/mL)	3926.6 [3715.9–5175.9]	3949.8 [3835.2–7273.1]	0.86
(ii) PCT levels (ng/mL)	0.5 [0.01–0.9]	0.3 [0.03–0.6]	0.53
(iii) CRP levels (mg/L)	4.1 [1.2–8.3]	6.2 [1.0–9.1]	0.07

Umbilical cord PTX3 levels (pg/mL)	2801.6 [3032.4–5085.9]	3890.3 [3374.4–4433.2]	0.13

Newborns			
(i) PTX3 levels (pg/mL)	6073.3 [4664.7–7481.9]	6630.9 [5396.0–7865.8]	0.14
(ii) PCT levels (ng/mL)	17.8 [9.9–25.6]	18.1 [7.8–28.5]	0.74
(iii) CRP levels (mg/L)	9.5 [1.1–17.9]	7.3 [1.2–13.5]	0.57

**Table 3:** Laboratory findings in the mothers' blood samples, umbilical cord blood samples, and neonatal blood samples in the study group (newborns with infection) and the control group. Serum pentraxin 3 (PTX3), C-reactive protein (CRP), and procalcitonin (PCT) levels as well as platelets and white blood count (WBC) are presented as a median and 95% confidence interval for median.

Investigated group	Study group (*n* = 29)	Control group (*n* = 47)	*p* value
Mothers			
(i) PTX3 (pg/mL)	4430.5 [2935.4–5925.6]	5343.8 [4265.2–6422.5]	0.06
(ii) CRP (mg/L)	5.5 [3.1–10.1]	4.7 [3.3–6.8]	0.65
(iii) PCT (ng/mL)	0.6 [0.4–1.2]	0.5 [0.1–0.9]	0.88
(iv) WBC (×10^3^/*μ*L)	12.3 [10.6–14.1]	11.3 [10.4–12.1]	0.68
(v) Platelets (×10^3^/*μ*L)	154 [128–174]	165 [135–184]	0.22

Umbilical cord PTX3 levels (pg/mL)	5077.4 [3737.2–6417.7]	3270.9 [2856.6–3685.1]	0.02

Neonates			
(i) PTX3 (pg/mL)	8455.8 [6339.2–10572.5]	4962.7 [4356.7–5568.8]	0.01
(ii) CRP (mg/L)	2.0 [1.0–7.8]	1.3 [0.7–2.2]	0.20
(iii) PCT (ng/mL)	27.7 [16.8–38.6]	11.8 [7.2–16.5]	0.02
(iv) WBC (×10^3^/*μ*L)	15.1 [13.0–17.7]	16.2 [14.2–19.1]	0.22
(v) Platelets (×10^3^/*μ*L)	221.4 [196.3–258.5]	254.8 [239.0–268.7]	0.11

**Table 4:** Correlations between pentraxin 3 levels and selected parameters in investigated newborns.

	Spearman's rho	*p* value
Birth weight	−0.16	0.18
Premature rupture of membranes	0.24	0.04
C-reactive protein levels	0.09	0.66
Procalcitonin levels	0.16	0.05

## Data Availability

The data used to support the findings of this study are available from the corresponding author upon request.
